# Optical Properties of Composites Based on Graphene Oxide and Polystyrene

**DOI:** 10.3390/molecules25102419

**Published:** 2020-05-22

**Authors:** Malvina Stroe, Mirela Cristea, Elena Matei, Andrei Galatanu, Liviu C. Cotet, Lucian C. Pop, Monica Baia, Virginia Danciu, Ion Anghel, Lucian Baia, Mihaela A. Baibarac

**Affiliations:** 1 Laboratory Optical Processes in Nanostructure Materials, National Institute of Materials Physics, Atomistilor str. 405 A, 77125 Bucharest, Romania; malvina@infim.ro (M.S.); mirela.cristea@infim.ro (M.C.); elena.matei@infim.ro (E.M.); gala@infim.ro (A.G.); 2 Department of Chemical Engineering, Faculty of Chemistry and Chemical Engineering, Babes-Bolyai University, Arany Janos str. 11, 400028 Cluj-Napoca, Romania; cotetcosmin@yahoo.com (L.C.C.); uklcp@yahoo.co.uk (L.C.P.); vir3riv@yahoo.com (V.D.); 3 Advanced Materials and Applied Technologies Laboratory, Institute of Research-Development-Innovation in Applied Natural Sciences, Babes-Bolyai University, Fantanele str. 30, 400294 Cluj-Napoca, Romania; monica.baia@phys.ubbcluj.ro (M.B.); lucian.baia@phys.ubbcluj.ro (L.B.); 4 Faculty of Physics, Babes-Bolyai University, M. Kogalniceanu str. 1, 400048 Cluj-Napoca, Romania; 5 Fire Officers Faculty, “Alexandru Ioan Cuza” Police Academy, Morarilor str. 3, 022451 Bucharest, Romania; ion_anghel2003@yahoo.com

**Keywords:** polystyrene microsphere, graphene oxide, composite, covalent functionalization

## Abstract

In this work, new optical properties of composites based on polystyrene (PS) microspheres and graphene oxide (GO) are reported. The radical polymerization of styrene in the presence of benzoyl peroxide, pentane and GO induces the appearance of new ester groups in the PS macromolecular chains remarked through an increase in the absorbance of the infrared (IR) band at 1743 cm^−1^. The decrease in the GO concentration in the PS/GO composites mass from 5 wt.% to 0.5 wt.% induces a diminution in the intensities of the D and G Raman bands of GO simultaneous with a down-shift of the D band from 1351 to 1322 cm^−1^. These variations correlated with the covalent functionalization of the GO layers with PS. For the first time, the photoluminescent (PL) properties of PS/GO composites are reported. The PS microspheres are characterized by a PL band at 397 nm. Through increasing the GO sheets’ concentration in the PS/GO composite mass from 0.5 wt.% to 5 wt.%, a PS PL quenching process is reported. In addition, in the presence of ultraviolet A (UVA) light, a photo-degradation process of the PS/GO composite having the GO concentration equal to 5 wt.% is demonstrated by the PL studies.

## 1. Introduction

In the last nine years, a sustained effort has been made to synthesize composites based on polystyrene (PS) and various carbon nanoparticles, such as multi-walled carbon nanotubes [1,2], single-walled carbon nanotubes [3], fullerene [3], reduced graphene oxide [4,5], graphene oxide (GO) [6], GO functionalized with phosphor-based organic compounds [7], and sulfonated GO [8]. The interest in these composite materials was reported to be for use as flame retardants [9] and non-volatile memory devices [10]. In the case of the PS/GO composites, the following six synthesis methods were used until now: (i) suspension polymerization [11], (ii) microemulsion polymerization [12], (iii) electrostatic self-assembly [13], (iv) reversible addition fragmentation chain transfer [14], (v) in situ polymerization followed by melt process [15], and Pickering emulsion polymerization [8]. Using X-ray photoelectron spectroscopy (XPS) and IR spectroscopy, the mechanisms proposed for the microemulsion and suspension polymerizations of styrene in the presence of GO were demonstrated to induce: (i) the opening of ether cycles on the GO surface [11] and (ii) the appearance of ester groups [12]. Using scanning electron microscopy (SEM) [11,12,15], transmission electron microscopy (TEM) [11,12,13,14,15] and X-ray diffraction (XRD) [11,12,13,14,15], the morphological and structural properties of the PS/GO composites were reported.

In order to obtain composites based on expandable PS and GO, in this work the attention was focalized on the radical polymerization of styrene in the presence of the GO sheets and pentane. The influence of the GO sheets’ weight on the PS spheres’ size was analyzed using SEM and dynamic light scattering (DLS). Using Raman scattering and IR spectroscopy, a polymerization mechanism of styrene in the presence of GO and pentane was reported. For the first time, the photoluminescence (PL) of the PS/GO composite was evidenced and the role of the GO sheets in the PS spheres’ PL was also reported. A photo-degradation process of the PS/GO composites with a GO sheets concentration of 5 wt.% is also highlighted in this work by PL.

## 2. Materials and Methods

Styrene, benzoyl peroxide (BPO), dimethylformamide (DMF), ethanol and benzene were purchased from the Sigma-Aldrich Company Ltd, Poole, UK and used without further purification. Other compounds were purchased from: (i) S.C. Nordic Invest S.R.L., such as H_2_SO_4_, H_3_PO_4,_ and HCl; (ii) S.C. “Hipocrate 2000” S.R.L., such as H_2_O_2_, (iii) Fluka, the graphite powder, and (iv) Merck, such as KMnO_4_.

The GO membranes were prepared by oxidative-exfoliation method [16,17]. In detail, 742 mL H_2_SO_4_ (95–97%, SC Nordic Invest SRL, Cluj-Napoca, Romania) was mixed by stirring with 83 mL H_3_PO_4_ (85%, SC Nordic Invest SRL, Cluj-Napoca, Romania) and then with 7.5 g graphite (powder, <0.1 mm, Fluka, (Leicestershire, England). Then, 33 g KMnO_4_ (99%, Merck, Darmstadt, Germany) was added slowly over the obtained suspension placed in an ice bath. After 2 days of ambient conditions, the reaction suspension was placed again by stirring in an ice bath and 550 mL of H_2_O_2_ (3%, SC “Hipocrate 2000” SRL, Bucharest, Romania) was slowly added. There followed steps of centrifugation (5000 rpm/10 min)-decantation-washing-sonication (15 min) in 550 mL H_2_O, 275 mL HCl (37%, SC Nordic Invest SRL, Cluj-Napoca, Romania) and 275 mL ethanol (absolute, SC Nordic Invest SRL, Cluj-Napoca, Romania). The last two processes were repeated two times. The obtained product was dispersed in 550 mL H_2_O and kept for 4 days in a sealed jar. After that, about 90% of the suspension volume from the upper part was harvested and spread onto a large glass support and placed for drying in open air under ambient conditions. GO as membranes (i.e., unsupported film) was obtained by mechanical exfoliation of the obtained GO film.

The radical polymerization of styrene (2 g) was started in the presence of benzene (40 mL) and BPO (0.04 g) at a temperature of 90 °C. The reaction was performed using a 1-L flat-bottom polymerization vessel equipped with a reflux condenser, mechanical stirrer, thermometer and two access inlets for nitrogen and pentane, respectively. A nitrogen bubbling was performed prior to the start of the polymerization reaction. In order to obtain expandable PS, four hours after the start of the polymerization reaction pentane was added to the reaction mixture (4 mL) as a foaming agent and the reaction was conducted at 120 °C for a further two hours. The polymerization reaction was stopped by adding about 500 mL ethanol when the temperature of the reaction mixture reached room temperature. Afterwards, filtration of the crude product and drying at a temperature of 60 °C were performed until a constant weight was achieved. Finally, a white powder of expandable PS was obtained.

With the intention of obtaining PS/GO composites, different GO amounts, i.e., 11, 22, 43, 66, 90 and 112 mg, were suspended in 10 mL DMF and added to the styrene mixture. Following all the above-mentioned steps in the case of expandable PS synthesis, PS/GO composites of gray color having a GO concentration equal to 0.5, 1, 2, 3, 4 and 5 wt.%. were obtained.

SEM pictures of GO membranes and PS/GO composites were recorded with a Zeiss Gemini 500 scanning electron microscope (Zeiss, Oberkochen, Germany).

The distribution of the PS spheres’ size in the case of compounds synthetized in this work was determined by a static light scattering method with Fritsch Analysette 22 NanoTec equipment from Fritsch GmbH, Idar-Oberstein, Germany, which has a measuring range from 10 to 2 mm. The light is obtained from two linear polarized lasers (one green, i.e., λ = 532 nm and another in IR, λ = 850 nm) and both forward and back-scattered signals are recorded by an array of detectors. For each sample, a background signal from the dispersion fluid (bi-distilled water) was recorded and then dispersions of the materials in water with an opacity of 10% were cycled five times through the particle size analyzer in order to obtain the best homogeneity. The last three measurement cycles provided virtually identical distributions. The values recorded from the fifth cycle are presented in this work.

Raman spectra of the GO membranes, the expandable PS and the PS/GO composites were recorded with a Raman spectrophotometer, T64000 model, from Horiba Jobin Yvon (Palaiseau, France) equipped with an AR laser (the excitation wavelength used was equal to 514 nm).

IR spectra of the GO membranes, the expandable PS and the PS/GO composites were recorded with a FTIR spectrophotometer, Vertex 80 model, from Bruker (Billerica, MA, USA).

PL and photoluminescence excitation (PLE) spectra of the expandable PS and the PS/GO composites were recorded with a Flurolog-3 spectrometer, FL3-22 model from Horiba Jobin Yvon (Palaiseau, France), under an excitation wavelength equal to 327 nm.

## 3. Results and Discussions

### 3.1. Optical Properties of the PS Microspheres and Their Composites with GO

#### 3.1.1. Morphological Properties of the PS Microspheres and Their Composites with GO

**Figure 1:**
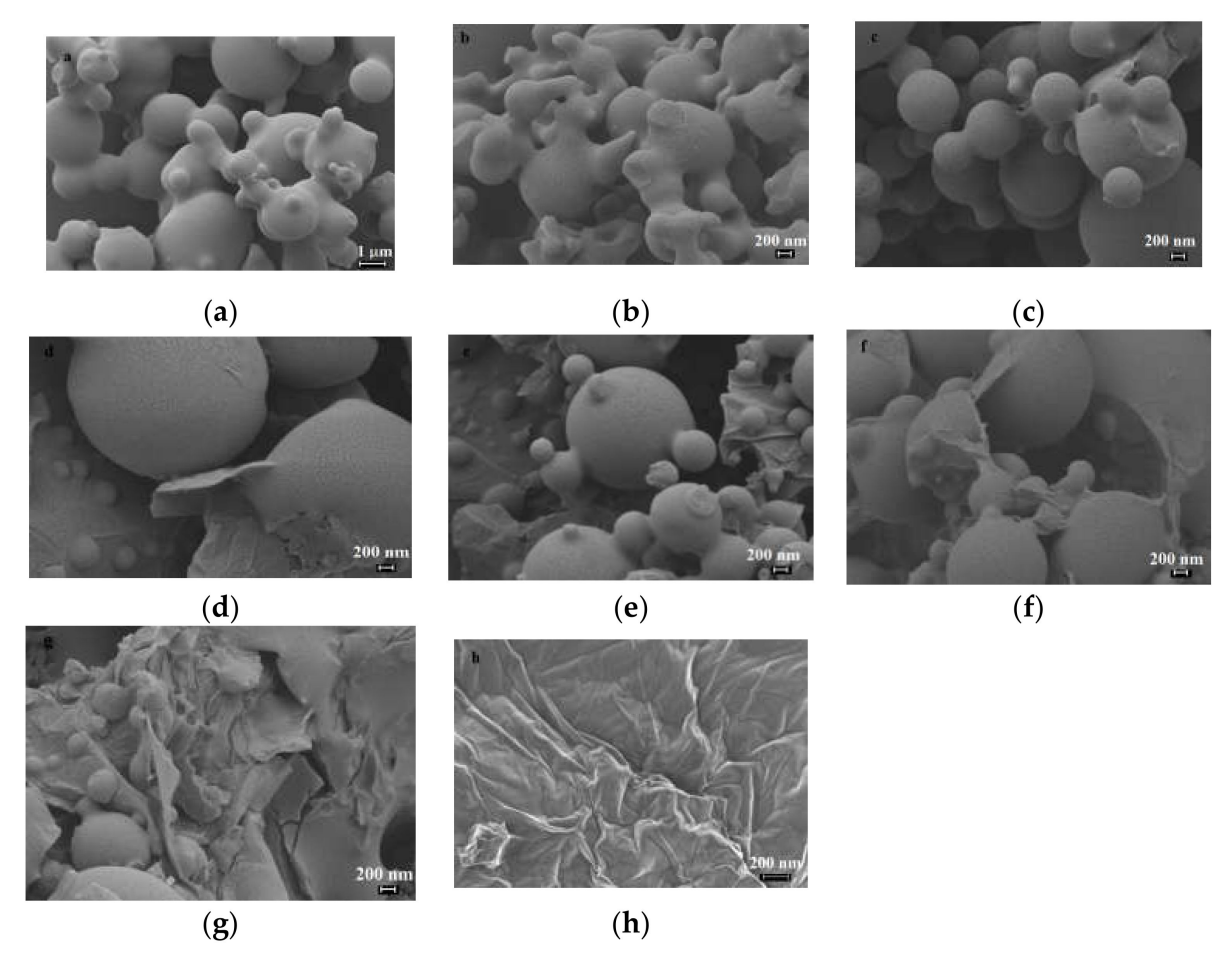
Scanning electron microscopy (SEM) pictures of the polystyrene/graphene oxide (PS/GO) composites have different GO concentrations equal to 0 wt.%. (**a**), 0.5 wt.% (**b**), 1 wt.% (**c**), 2 wt.% (**d**), 3 wt.% (**e**), 4 wt.% (**f**) and 5 wt.% (**g**). Subfigure (**h**) shows the SEM picture of the GO membrane.

Figure 1 shows the SEM pictures of the expandable PS, GO membranes and PS/GO composites.

**Figure 2:**
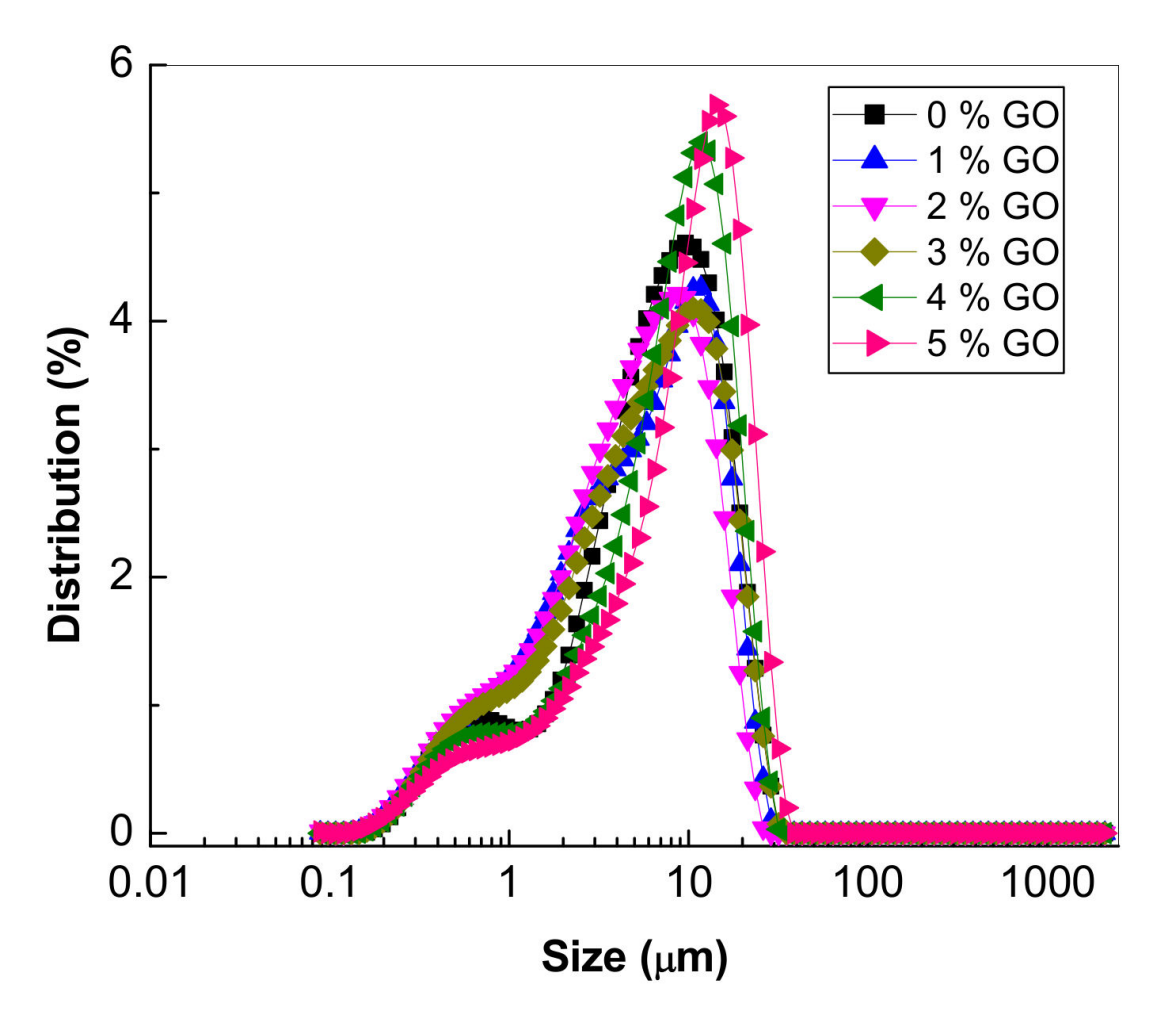
Particle size distribution plots of the PS/GO composites having a GO concentration equal to 0 wt.% (black curve), 0.5 wt.% (red curve), 1 wt.% (blue curve), 2 wt.% (magenta curve), 3 wt.% (olive curve), 4 wt.% (green curve) and 5 wt.% (pink curve).

The adopted synthesis method in this work for the expandable PS leads to spheres with diameters of between 300 nm and 3.5 µm being obtained (Figure 1a). Adding the GO sheets suspension in the mixture of the radical polymerization reaction of styrene induces an adsorption of the GO sheets onto the PS spheres’ surface as shown in Figure 1b–g. With a higher concentration of the GO sheets, around 4 and 5 wt.%, a wrapping of the expandable PS spheres is observed to occur. The PS particles’ size distribution depending on the GO sheets concentration in PS/GO composite mass is shown in Figure 2. With a higher concentration of the GO sheets (i.e., 4 and 5 wt.%), the wrapping the PS spheres leads to an increase in the size of these particles and in this sense the green and pink curves in Figure 2 are relevant.

#### 3.1.2. Vibrational Properties of the PS Microspheres and Their Composites with GO

In order to confirm that these morphological structures belong to the expandable PS and the PS/GO composites, the vibrational properties of these compounds were investigated by Raman scattering and IR spectroscopy.

**Figure 3:**
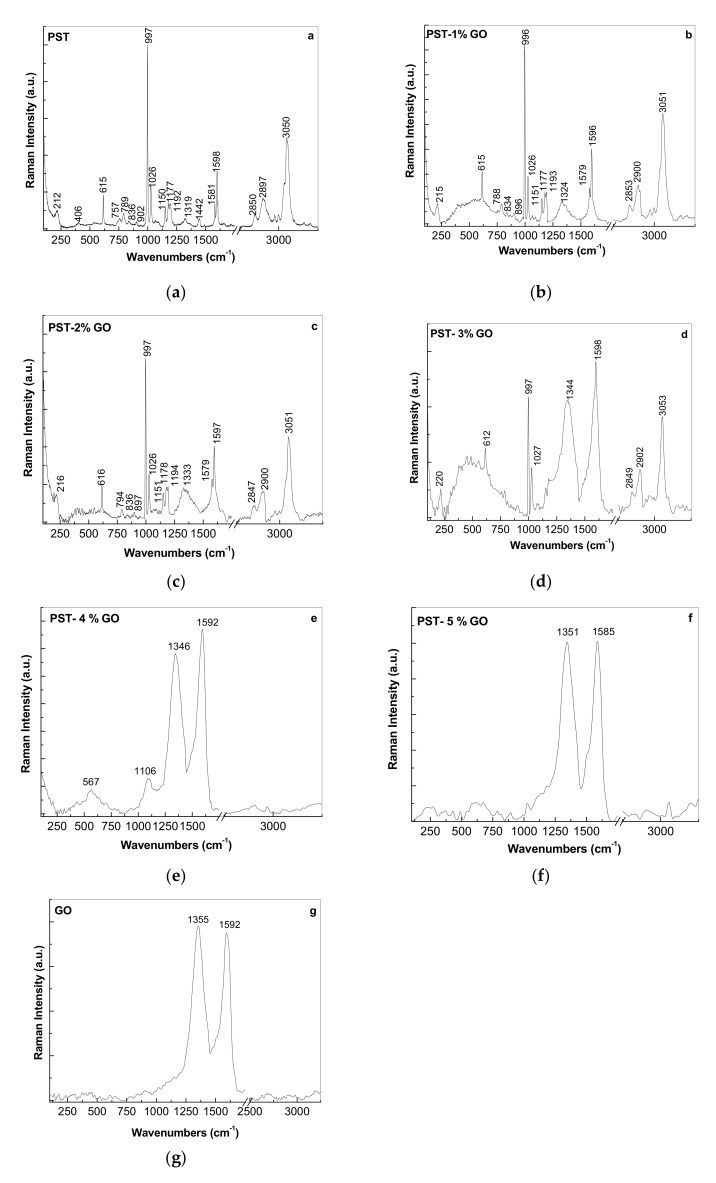
Raman spectra of the PS/GO composites having a GO concentration equal to 0 wt.% (**a**), 1 wt.% (**b**), 2 wt.% (**c**), 3 wt.% (**d**), 4 wt.% (**e**) and 5 wt.% (**f**). In (**g**), the Raman spectrum of the GO sheets is shown.

According to Figure 3a, the main Raman lines of the PS spheres are situated at 615, 758, 795, 996, 1028, 1151-1177-1195, 1326, 1448, 1579-1598, 2850-2906 and 3053 cm^−1^ and their assignment is shown in Table 1 [18,19,20].

In the 200–3500 cm^−1^ spectral range, the Raman spectrum of the GO sheets is dominated by two bands peaked at 1355 and 1592 cm^−1^. The first one, often labeled as D band, is assigned to the radial vibration mode of hexagonal carbon cycles, whose intensity increases with the number of defects induced in the graphitic network as a consequence of the presence of carbon atoms with sp^3^ hybridization [16,21]. The second band, labeled as G band, is assigned to the E_2g_ mode at the center of the Brillouin area [16,21]. The ratio between the relative intensities of the D and G bands (I_D_/I_G_) is equal to 1.04. The main changes induced in the Raman spectra by the GO presence shown in Figure 3 are: (i) the change from 3.22 to 2.38, 2.16 and 0.77 of the value of the ratio between the intensities of the Raman lines peaked at 997 and 1598 cm^−1^, when the concentration of the GO sheets in the PS matrix increases from 0 to 1, 2 and 3 wt.%, respectively; (ii) an up-shift of the D band from 1324 cm^−1^ to 1333, 1344, 1346 and 1351 cm^−1^, when the GO sheets concentration is changed from 1 wt.% to 2, 3, 4 and 5 wt.%, respectively. These changes are accompanied by a gradual increase in the intensity of the Raman line at 1579 cm^−1^ versus the Raman line peaked at 1595 cm^−1^; (iii) the diminution of the ratio between the intensities of the Raman lines having maxima at 997 and 3050 cm^−1^ (I_997_/I_3050_) from 2.00 to 0.77, when the GO sheets concentration in the PS matrix is equal to 0 and 5 wt.%, respectively. These changes indicate that as the GO sheets concentration is increasing, the generation of the PS oligomers takes place.

**Figure 4:**
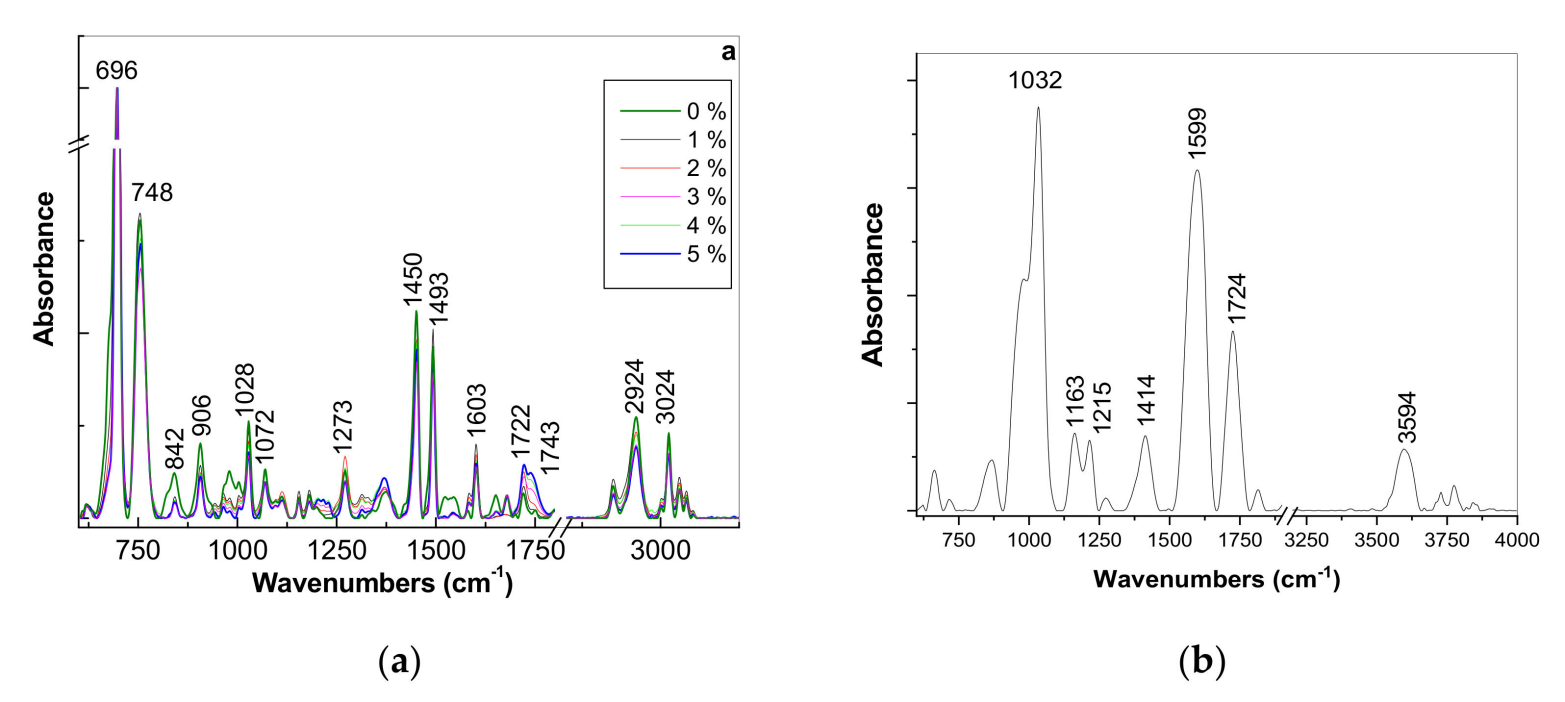
Infrared (IR) spectra of the PS/GO composites (**a**) having a GO concentration equal to 0 wt.% (dark green curve), 1 wt.% (black curve), 2 wt.% (red curve), 3 wt.% (magenta curve), 4 wt.% (open green curve) and 5 wt.% (blue curve). The IR spectrum of the GO sheets (**b**).

In order to obtain additional information, the IR spectra of the PS and its GO composites were recorded and are illustrated in Figure 4. The main absorption IR bands of the GO sheets as well as their assignment are shown in Table 2 [21].

**Scheme 1:**
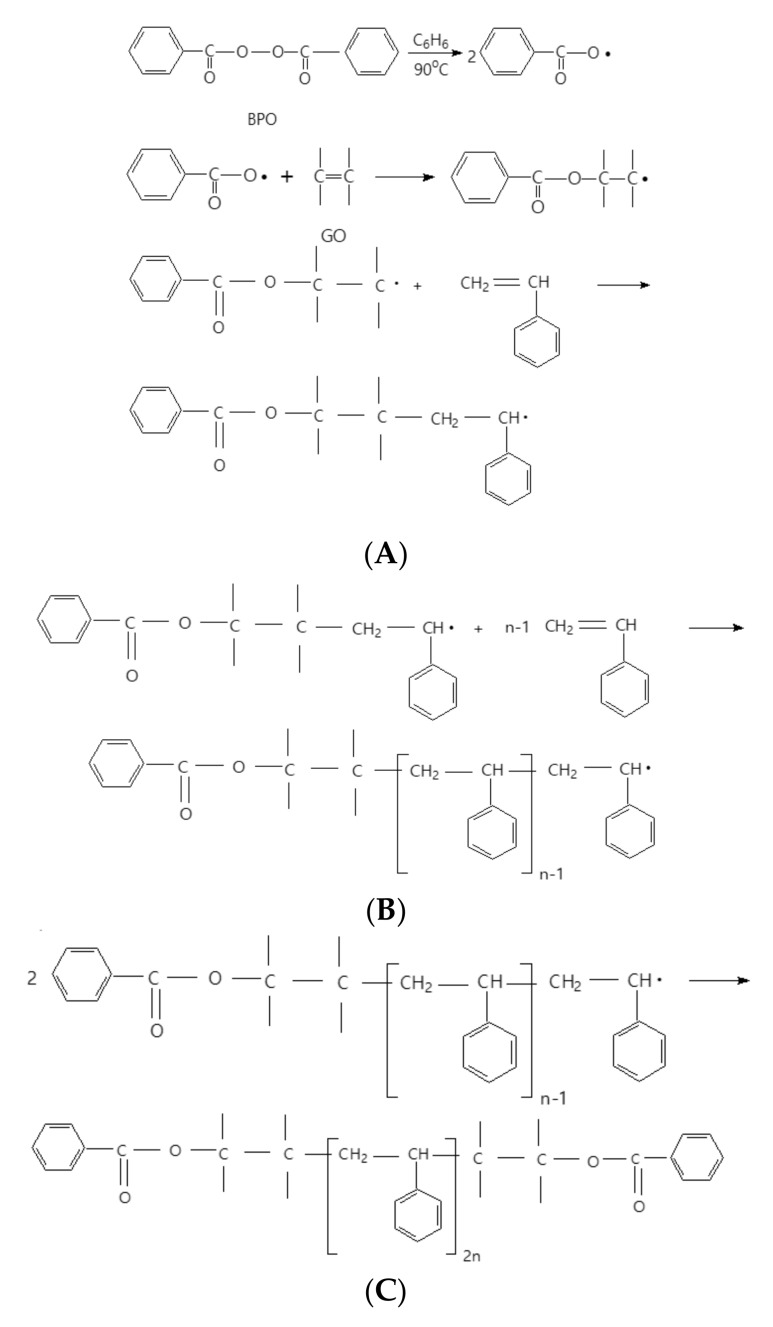
Chemical mechanism of the radical polymerization of styrene in the presence of the GO sheets: (**A**) The initiation reaction. (**B**) The propagation reaction of the macromolecular chain. (**C**) The macromolecular chain termination reaction. The reaction product corresponds to the GO sheets covalently functionalized with PS.

In the case of PS, the main absorption IR bands are peaked at 696- 748, 842-906, 982, 1028, 1072, 1244–1273, 1450, 1493, 1603, 1722, 1743, 2924 and 3024 cm^−1^, with these being assigned to the subsequent vibrational modes: C-H out-of-plane bending of C_6_H_6_ mono-substituted; amorphous phase; ring out-of-plane deformation; amorphous portion of PS; aromatic C-H out-of-plane bending; ring out-of-plane deformation; ring in-plane CCH bending; chain stretching; vinylidene C-H; ester group in C_6_H_5_COO-CH_2_-CH_2_-C_6_H_5_-belonging to PS; ester group in C_6_H_5_COO-C-C-belonging to the GO sheets; CH_2_ asymmetric stretching; and CH asymmetric stretching [6,16,22,23,24,25,26,27]. When increasing the GO sheets concentration in the PS matrix, one observes a gradual increase in the absorbance of the IR band peaked at 1743 cm^−1^. This fact indicates that the C_6_H_5_COO-bonding onto the GO sheets’ surface is induced. Taking into account the results shown in Figure 3 and Figure 4, the chemical mechanism of the radical polymerization of styrene in the presence of the GO sheets and BPO can be described according to reactions shown in Scheme 1.

#### 3.1.3. Photoluminescence of the PS Microspheres and Their Composites with GO

**Figure 5:**
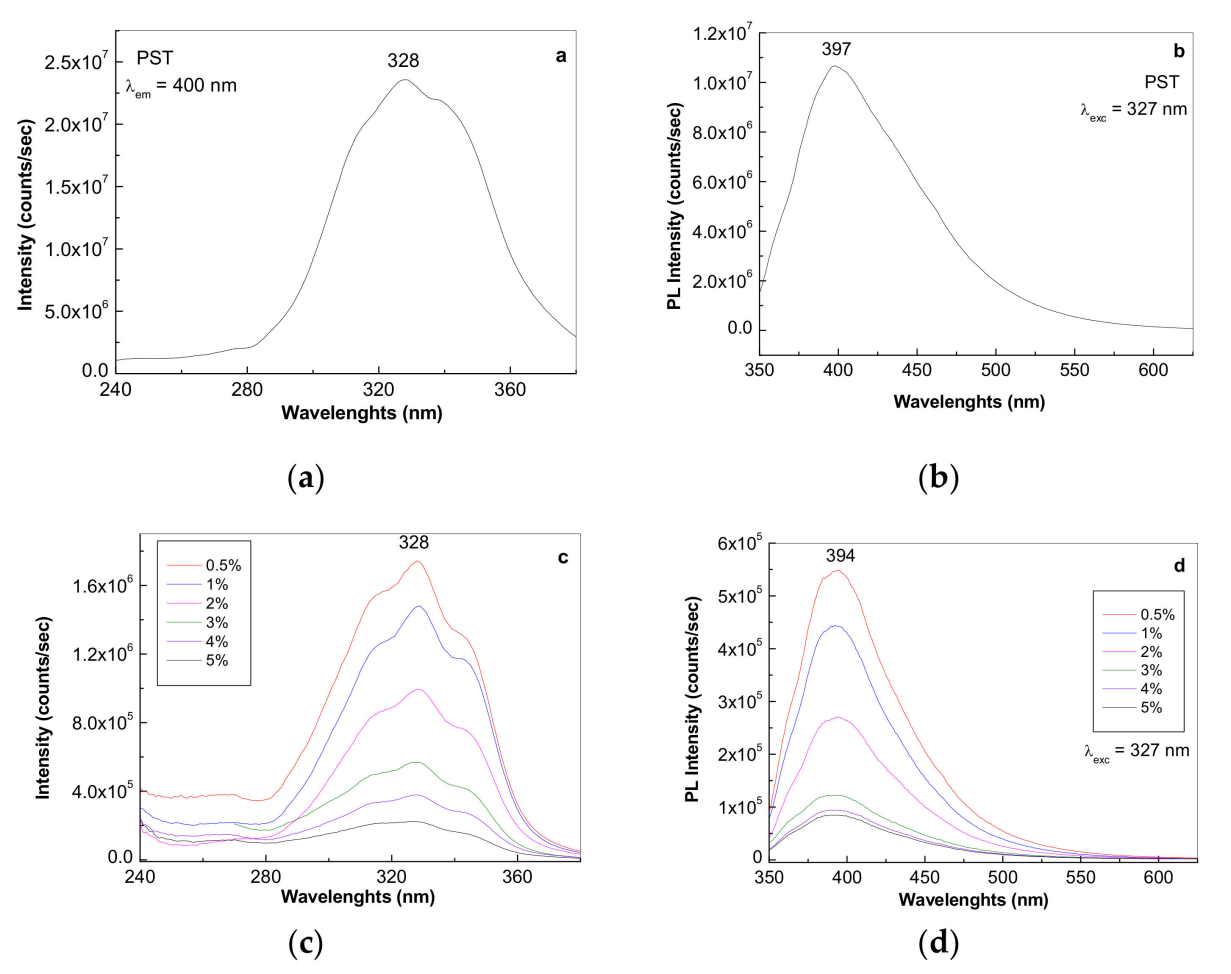
Photoluminescence excitation (PLE) and PLE spectra of PS (**a**,**b**) and the PS/GO composites (**c**,**d**) having a GO sheets concentration equal to 0.5 wt.% (red curve), 1 wt.% (blue curve), 2 wt.% (magenta curve), 3 wt.% (green curve), 4 wt.% (violet curve) and 5 wt.% (black curve).

The covalent functionalization of the GO sheets with PS induces significant changes in the PLE and PL spectra of the macromolecular compound. Figure 5 is relevant in this context.

**Figure 6:**
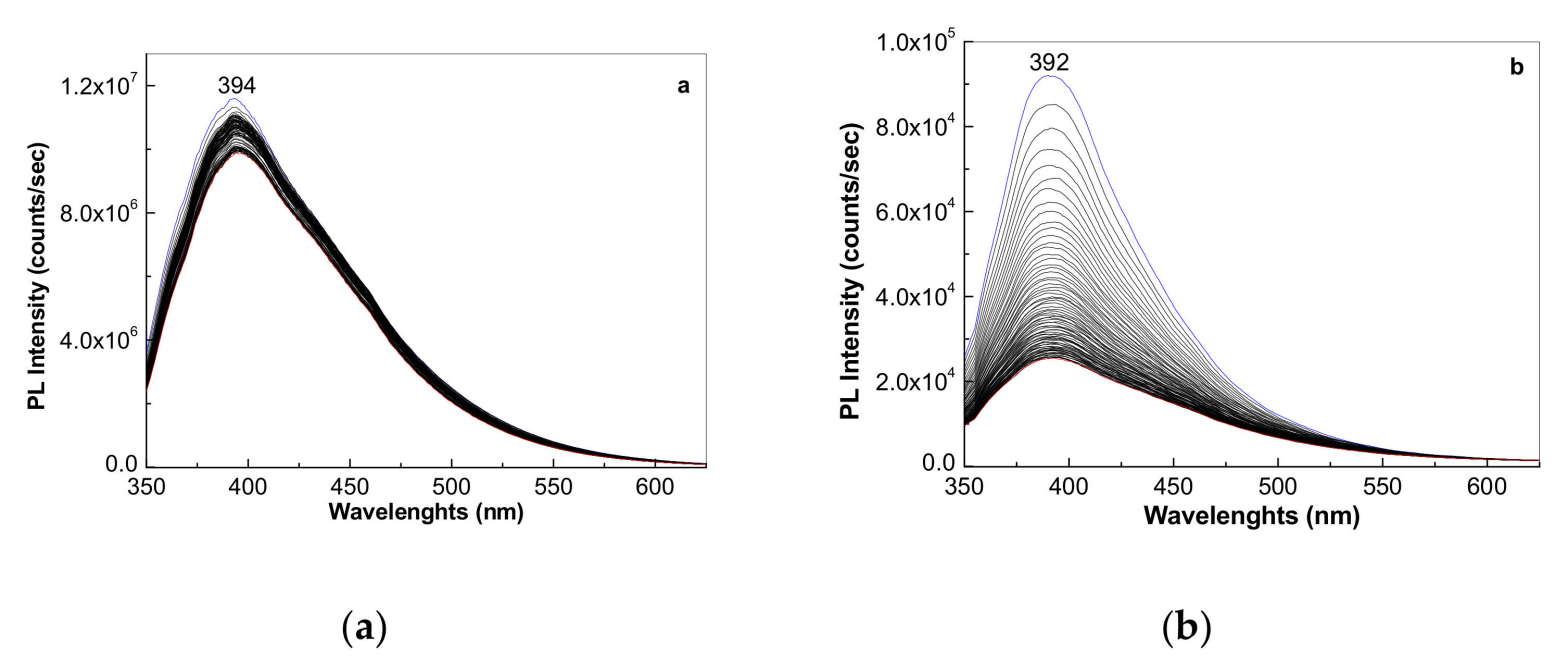
Photoluminescence (PL) spectra of PS (**a**) and the PS/GO composite having a GO sheets concentration equal to 5 wt.% (**b**) under UV light exposure for 3 h. The PL spectra of the two samples in the initial and final state correspond to the blue and red curves. Black curves correspond to intermediate PL spectra.

The PLE and PL spectra of the expandable PS are characterized by bands with maxima at 328 and 397 nm, respectively. One can remark a gradual decrease in the intensity of the PLE and PL spectra of the expandable PS, from 2.35 × 10^7^ and 1.06 × 10^7^ counts/s to 2.21 × 10^6^ and 8.45 × 10^4^ counts/s, when the GO sheets concentration in the PS/GO composite was increased from 0 wt.% to 5 wt.%. In our opinion, this PS PL quenching process originates in the new luminescent centers generated during the radical polymerization of styrene, when a covalent functionalization of the GO sheets with PS took place. As observed in Figure 6, significant changes are induced to new luminescent centers in the presence of UVA light. In comparison with PS (Figure 6a) or its composites with the GO sheets having a concentration of between 0.5 and 4%, in the case of the PS/GO sample with a concentration of 5 wt.% GO (Figure 6b), under the excitation wavelength of 327 nm, one observes a decrease in the PL spectrum intensity, from 9.18 × 10^4^ counts/sec to 2.55 × 10^4^ counts/s as the exposure time at UVA light increases to 3 h and 30 min. This behavior is difficult to explain at this stage of our investigations. However, we assume that this behavior originates in the appearance of defects on the PS macromolecular chain by the generation of new bonds such as -CH=C-(C_6_H_5_)-.

**Scheme 2:**
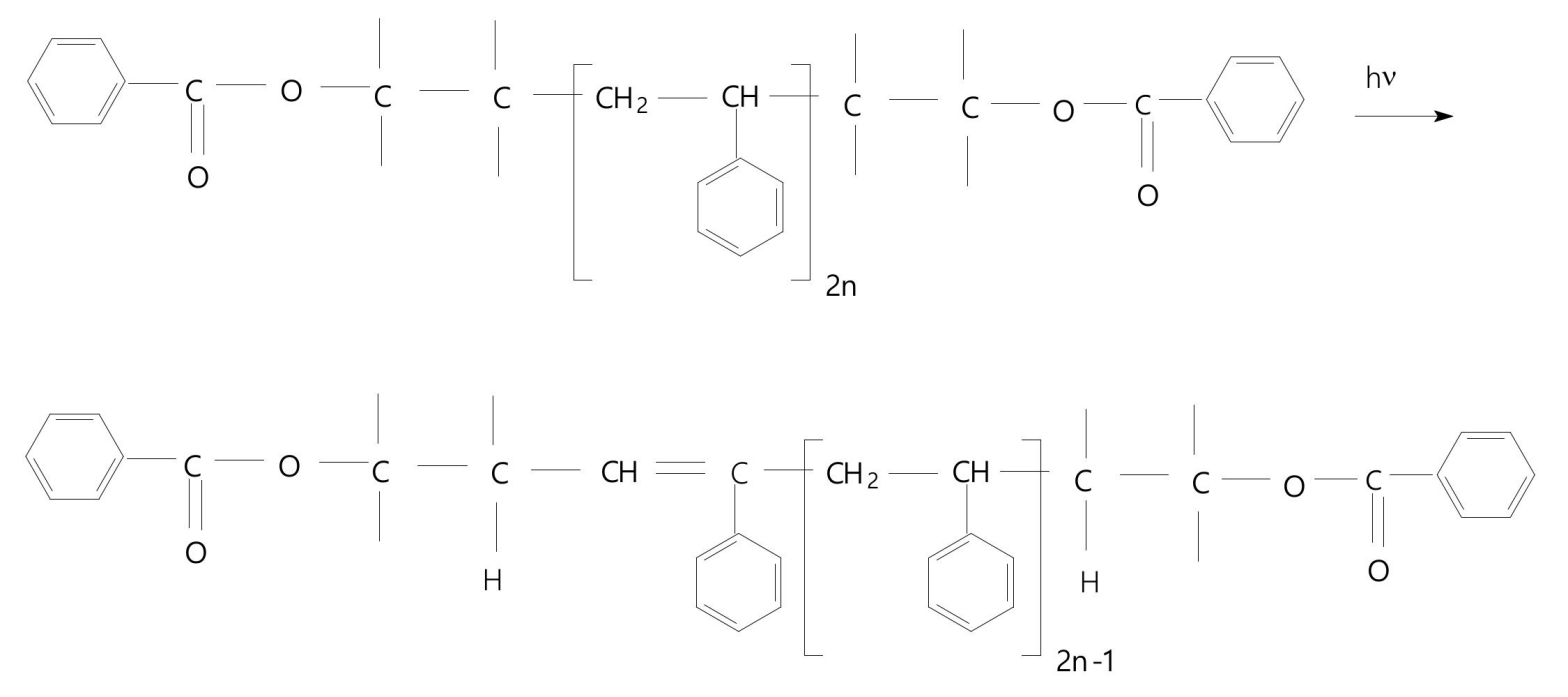
The photo-degradation reaction of the PS/GO composite under exposure to UVA light.

Such a photo-degradation process of the expandable PS/GO composite can be described by the reaction shown in Scheme 2.

**Figure 7:**
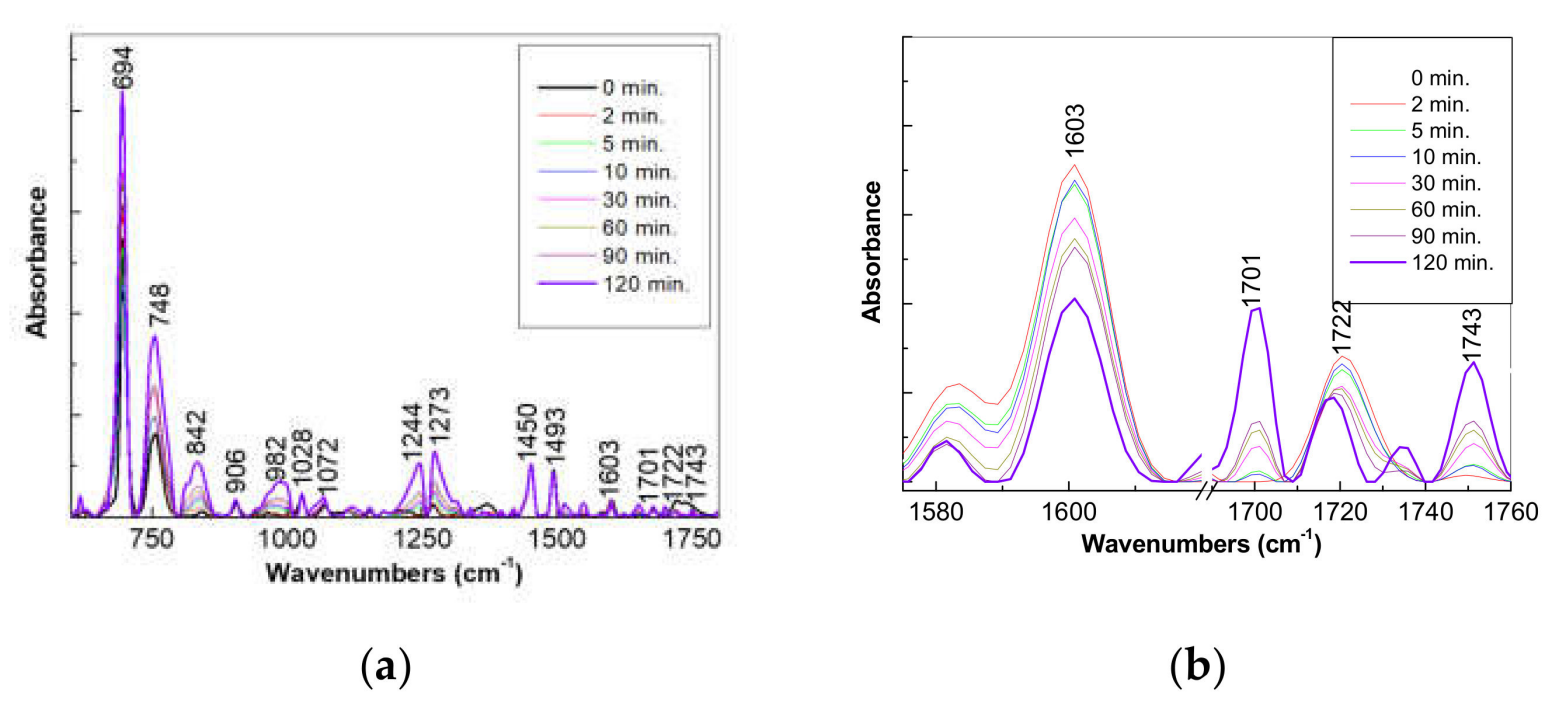
(**a**) IR spectra of the PS/GO composite having a GO sheets concentration equal to 5 wt.% under UV light exposure, time of 0 min (black curve), 2 min (red curve), 5 min (open green curve), 10 min (blue curve), 30 min (magenta curve), 60 min (dark green curve), 90 min (purple curve) and 120 min (violet curve). In (**b**), the spectral range 1560–1760 cm^−1^ of the IR spectra shown in (a) is detailed.

In order to support the photochemical reaction proposed in Scheme 2, Figure 7 shows the IR spectra of the PS/GO composite having a GO sheets concentration equal to 5 wt.% before and after 2, 5, 10, 30, 60, 90 and 120 min of UVA light exposure. When the UV exposure time increased to 120 min (i) an increase in absorbance of the IR bands with maxima at 837, 1244 and 1273 cm^−1^; (ii) a change of the ratio between the absorbance of the IR bands peaked at 1450 and 837 cm^-1^ from 5 to 1.7; (iii) an up-shift of the IR band from 1743 cm^−1^ to 1747 cm^−1^; and (iv) the appearance of a new IR band at 1701 cm^−1^ were observed. This IR band is situated close to the IR band at 1683 cm^−1^, reported to be assigned to the vibrational mode C=C stretching of the styrene vinyl group [28]. In our opinion, these variations support the appearance of the amorphous portions of PS and the modification of the GO sheets covalently grafted with BPO.

## 4. Conclusions

This work reports new results concerning the optical properties of the composites based on GO sheets and expandable PS. The radical polymerization of styrene in the presence of pentane and GO sheets leads to the GO sheets covalently functionalizing with PS. This functionalization process is confirmed by: (i) the down-shift of the Raman band from 1351 to 1322 cm^−1^ and (ii) the progressive increase in absorption of the IR band at 1743 cm^−1^. A PS PL quenching process is reported to take place in the presence of the GO sheets. Under UVA light exposure, a photo-degradation process of the PS/GO composite having a GO concentration equal to 5 wt.% has been reported. New evidence for the reaction proposed for the photo-degradation of the PS/GO composite is provided by IR spectroscopy. The possible practical applications of the PS/GO composites with structural and optical properties, anticipated for the next period, can be in the field of construction materials and sensors for different toxic gases such as NH_3_, NO_2_, CO and SO_2_. In this context, we highlight that the most recent developments concerning (i) sensors for NO_2_ using polystyrene beads decorated with graphene were reported by H. Fei et al. [29]; additionally, (ii) the flame retardants for the expandable PS, of the phosphorous-based compound-modified GO sheets type, were synthetized by Y.Z. Wang et al. [30].

## Figures and Tables

**Table 1:** Experimental vibrational wavenumbers (ν, cm^-1^) in the Raman spectra of the polystyrene (PS) spheres.

ν, cm^−1^	Assignment of Vibrational Modes [18,19,20]
615	out-of-plane benzene (B) ring deformation
758	CC stretching + ring CC stretching + ring trigonal + asymmetric deformation
795	out-of-plane C-H bending in mono-substituted B ring
996	in-plane bending C-H rocking
1028	C-H bending tangential E_1u_
1151-1177-1195	CC stretching + tangential C-H bending B_2u_
1326	C-H bending mode A_2g_ + C-O stretching
1448	C-H bending mode in ethylene (B_3u_)
1579–1598	C=C stretching
2850–2906	C-H stretching mode on the polymer chain
3053	radial C-H stretching mode E_1u_

**Table 2:** Experimental vibrational wavenumbers (ν, cm^-1^) in the IR spectra of the graphene oxide (GO) sheets.

ν, cm^−1^	Assignment of Vibrational Modes [21]
1032	C=C stretching mode
1163	C-O vibrational mode in ether group
1212	OH group at the GO ends
1414	the deformation mode of OH in the COOH group
1599	H_2_O adsorbed on the GO surface
1724	C=O stretching mode in COOH/CHO
3594	OH stretch vibration in the free H_2_O molecules
